# P81 Workforce development for antimicrobial stewardship: building pharmacy technicians’ awareness, confidence, and capability

**DOI:** 10.1093/jacamr/dlag102.087

**Published:** 2026-06-26

**Authors:** Sandeep Rai, Sarah Newsome, Alisha Lakha, Amy Laflin, Philip Jones, Abigail Stirling, Sejal Parekh, Vikki Connell, Amy John, Diane Ashiru-Oredope

**Affiliations:** UK Health Security Agency, London, UK; NHS England, UK; NHS England, UK; Association of Pharmacy Technicians, UK; Association of Pharmacy Technicians, UK; Sunlight Group Practice, Wirral, UK; NHS England, UK; University of Hospital North Midlands NHS Trust, UK; Prince Charles Hospital, Wales; UK Health Security Agency, London, UK

## Abstract

**Background:**

National survey findings (2019 and 2024) have identified gaps in antimicrobial resistance (AMR) knowledge among pharmacy technician respondents. This presents an opportunity to strengthen education, training and capability across the workforce. Two AMR webinars were developed and delivered in collaboration with the Association of Pharmacy Technicians UK (APTUK) and NHS England, supported by pre-knowledge surveys to assess baseline understanding and learning.

**Objectives:**

To assess pharmacy technicians’ knowledge, behaviours and practices related to AMR, and to explore their motivation to take action.

**Methods:**

An anonymized, consent-based survey was provided to participants via Microsoft Forms prior to delivering two national AMR webinars in March 2026. The questionnaire included items from a validated national AMR survey. Post-webinar reflections on key learning points were collected using a Slido feedback form. All responses were analysed using Microsoft Excel.

**Results:**

One hundred and fifty-nine (*n*=159) pharmacy technicians responded with 34% working in specialist roles (AMR and non-AMR roles), 46% in generalist roles and 19% pre-registration trainee pharmacy technicians. Hospital settings accounted for the largest proportion of respondents (40%), with community pharmacy representing 23%. Over half of participants (55%) reported no current involvement in AMR activities. Motivation to engage in antimicrobial stewardship (AMS) activities was attributed to professional responsibility (21%), a desire to develop further knowledge and skills (21%) and wanting to contribute to reducing AMR (19%). Only 8% of respondents felt their workplace encouraged involvement in AMS. Less than half the respondents (43.5%) had undertaken AMR/AMS specific education. Perceived understanding of AMR was reported at 94.3% whilst reported confidence in having sufficient antibiotic use knowledge for their role was 67.1%. Less than half (43%) felt they had a key role in controlling AMR. Half of the respondents 50.3% (80/159) answered all seven knowledge questions correctly. Almost all respondents recognized that antibiotics are ineffective against viruses (95.6%) and colds or flu (96.2%) and understood risks associated with unnecessary antibiotic use (98.7%). More than three quarters of participants were aware that all antibiotic use increases the risk of resistant infection (76.6%) and that healthy individuals can carry resistant bacteria (77.8%) Awareness that resistant bacteria can spread between people was the question with the lowest correct response rate of 62.7%. Key themes emerging from the written feedback post-webinar include an increased awareness of AMR and a greater familiarity with available AMS resources. Participants recognized the expanding role of pharmacy technicians within AMS, particularly in supporting public education. Many reported feeling motivated to implement changes in their practice, take practical stewardship actions, enhance patient education, and pursue further learning.

**Conclusions:**

Although knowledge levels were higher compared with previous national surveys in 2019 (40%) and 2024 (37.2%), this study demonstrates that increased knowledge does not translate into confidence, role ownership, or routine engagement in AMS activities. Targeted educational support is needed to build confidence, strengthen capability into day-to-day practice and support pharmacy technicians’ role within multidisciplinary teams. Future planned activities including focused role modelling articles in APTUK newsletters, peer-led videos, and dissemination of findings to mobilize knowledge and increase engagement.

